# French Students with Dyslexia Facing the Punctuation System: Insecurity, Inventory Use, and Error Studies

**DOI:** 10.3390/brainsci13040532

**Published:** 2023-03-23

**Authors:** Audrey Mazur, Matthieu Quignard

**Affiliations:** 1 Laboratoire d’Excellence ASLAN, University of Lyon, 69008 Lyon, France; 2 Laboratoire CNRS Interactions, Corpus, Apprentissages, Représentations, UMR 5191, 69007 Lyon, France

**Keywords:** dyslexia, higher education, written production, spelling

## Abstract

Punctuation strongly contributes to the cohesion of the text. Despite this relevant role in written activity, this linguistic paradigm is too rarely observed. Moreover, it is all the more important to analyze its management as it is one of the difficulties declared by students with dyslexia. In that sense, the purpose of this paper is to analyze punctuation management during written text production by students with dyslexia, compared to matched control students. Previous English and Dutch studies confirm this feeling and reveal that students with dyslexia make many punctuation errors. That being said, there is no consensus; other studies do not reach this conclusion. For this present study, students with dyslexia and control students matched in age, university level, and gender were asked to produce spontaneous written and spoken narrative and expository texts. The written texts (N = 86) were collected using Eye and Pen© software with digitizing tablets. Results reveal that if students with dyslexia use the same inventory of punctuation marks as control students, they use fewer punctuation marks and make more errors than control students. These results are discussed and highlighted by the literature dealing with written production as a complex cognitive activity. They reveal that punctuation management is deficient for students with dyslexia, suggesting that the cohesion system can be impacted by dyslexia.

## 1. Introduction

### 1.1. Definition of Dyslexia

Our society places special emphasis on normative and prototypical writing, the management of which is said to increase “the chances of integration and even ascent in the social ladder” [1]. Thus, we can understand the interest in late language development [2], especially for individuals with specific learning disabilities. The DSM-5 [3] classifies dyslexia as one of the seven categories of neurodevelopmental disorders, namely, specific learning disabilities. This category encompasses all symptoms of reading, writing, and calculation disorders as defined by the diagnostic criteria listed below. Specific learning disabilities would involve skills significantly below those expected for a given chronological age, interfering negatively and significantly with school or university academic performance. The most common manifestation of specific learning disabilities is dyslexia, a term that refers to “a learning profile characterized by difficulties in recognizing common words accurately or fluently and poor decoding and spelling skills” ([4], p. 2). Since the Ringard report [5], reporting a significant prevalence of dyslexia (6–8%) [6], students are better detected and treated. Thanks to earlier diagnoses and the implementation of remediation from an early age, more and more individuals with dyslexia are entering higher education, developing reading and writing strategies specific to them, and allowing them to manage “intensive exposure to the written word” ([7], p. 1). That being said, great difficulties remain: the academic progress and success that dyslexic students can achieve in no way eliminates dyslexia or even its manifestations. Dyslexia in higher education is also a topical and public health issue. 

### 1.2. Dyslexia in Higher Education in France

Indeed, higher education institutions are welcoming an increasing number of students with disabilities, in the United Kingdom [8,9], for instance, or in France [10]. In France, for the 2020–2021 academic year, 39,786 students enrolled in higher education are reported as having a disability, which represents 1.82% of students and an increase of 6.1% from the previous academic year. The Ministry of Higher Education, Research, and Innovation mentions that this represents a 12.1% increase in students with disabilities per year since 2005 [11]. Among them, 34,250 students are at university, and 8478 are declared to have speech and language impairments, which represents 24.8% of students with disabilities ([11], p. 39, Figure 5). This number may hide a much more concerning reality. Indeed, in 2015 and 2016, two surveys on dyslexia in higher education were conducted at the University of Lyon. The objective was to understand the needs and difficulties of students with dyslexia at the University of Lyon (for a presentation of several results, see [12,13]). Various questions concerning different topics were asked about student life, learning, reading, writing, or diagnosis. One of the questions was, “Are you declared to the disability services of your institution?” For both questionnaires, approximately the same result was obtained: almost half of the students with dyslexia were not declared to the institution’s disability service at their university [14]. That means that, potentially, one out of every two students with dyslexia is not declared to their institution’s disability service. Thus, the number of students with dyslexia in higher education may be higher than reported. 

### 1.3. Persistent Difficulties and Insecurity of Dyslexia Students Regarding Punctuation

Moreover, analyses of these questionnaires at the university of Lyon (previously mentioned; you can see [12,13] for a presentation of results) reveal that the written task most complicated for students with dyslexia is the production of essays (whereas control students do not feel this difficulty as much; Chi2 = 11.3, *p* < 0.05) and the production of a dissertation (whereas control students do not feel this difficulty as much; Chi2 = 9.6, *p* < 0.05). Moreover, students with dyslexia declare having much difficulty in the organization of their written texts (more than control students; Chi2 = 50.4, *p* < 0.001), with the agreement and tense of verbs (more than control students; Chi2 = 32.6, *p* < 0.001), they report fear of making spelling mistakes (more than control students; Chi2 = 33.9, *p* < 0.001), and a deficit in vocabulary (more than control students; Chi2 = 8.4, *p* < 0.05) [12,13]. These feelings are confirmed by the experimental analyses that follow these questionnaires. For instance, they make more errors of spelling and verb agreement than control students (for instance, [14,15]). Moreover, these findings are consistent with previous international studies. English students with dyslexia have difficulties with written production, whether in terms of the number of polysyllabic words [16], the number of spelling errors [16,17,18], certain syntactic aspects [16], or the identification of errors and their correction [16,17,19]. Furthermore, they also experience difficulty revising their text, with less effective textual revision compared to student controls [12,16,20,21].

One of the other difficulties mentioned by students with dyslexia is punctuation. In the questionnaires mentioned above [12,13,20], we asked students if they have difficulties using punctuation marks; students with dyslexia seem to have more difficulties with these marks than control students (Chi2 = 19.9, *p* < 0.001): they have systematically (9.8%, control student 1.2%), frequently (15.9%, control student 12.2%), or sometimes (31.7%, control student 12.2%) experienced difficulties in handling punctuation. Control students report little difficulty with punctuation (74.4%, students with dyslexia, 42.7%). This is not the most important difficulty in writing that dyslexic students complain about, but it is mentioned, and the international literature about dyslexia confirms that punctuation is problematic for people with dyslexia [22,23,24,25]. Dyslexic students probably talk less about this aspect of writing than spelling, for example, because it is a poor relation to written production: punctuation is not explicitly taught in school [26,27,28,29]. In France, education about punctuation at school is restricted to periods, commas, and capitals. Nevertheless, some previous studies reveal the important function of punctuation in the organization and structuration of a written text (among others, [28,30,31,32,33,34,35]). Moreover, in a previous study concerning the lexical choices of students with dyslexia [14], our analyses revealed that one of the general indicators was the number of punctuation marks. This indicated that there is a significant difference in the proportion of punctuation marks: students with dyslexia use less punctuation than control students. 

In this present study, we propose to analyze the use and management of punctuation by dyslexic students in written texts. The performances of 21 students with dyslexia and 22 non-dyslexic students matched in gender, age, and school level were analyzed. This paper provides information about the difficulties of students with dyslexia in writing and aims to contribute to the literature concerning dyslexia in adulthood. Indeed, studies on dyslexic adults are still too few, and it is necessary to overcome this deficiency in French and international research [7,35,36,37]. Moreover, it is reported that, over a period from 2000 to 2019, only 605 studies (i.e., 28.8%) were conducted on adults with dyslexia. The authors [37] group these studies into three main categories: clinical, cognitive, and neuroscience. Studies on adults with dyslexia are rare, and those on the impact of dyslexia on language production, which it directly affects, do not seem to be listed. Indeed, comparisons between a group of students with dyslexia and a matched control group that observes their writing skills during written text production are relatively uncommon [38]. This present study can also complete the existing ones from a psycholinguistic perspective. We aim to use our results to guide remediation by providing keys to understanding difficulties in spelling.

## 2. Written Production, Punctuation, and Dyslexia

### 2.1. Written Production: A Complex Task

The activity of writing is one of the most costly and complex cognitive tasks (among others [39,40,41,42,43,44]), involving various cognitive processes such as text interpretation, reflection, the generation of ideas and linguistic structures, problem solving (including planning), decision making, and the production of inferences [40]. The main cognitive processes identified in the composition task are planning, translating, and reviewing [41]. Planning allows for the generation of ideas while relating them to the specific context of the text’s production. Translating allows for the development of internal representations in linguistic and graphic structures. And finally, reviewing is a matter of performing control operations on the text. Other processes come into play, such as problem solving (including planning), decision making, and the production of inferences [40], all of which are carried out with the help of long-term memory and the task environment. This model, which was intended to account for written production in the adult expert, has been the subject of numerous elaborations, which have resulted in a version that allows for the progressive construction of writing expertise and can be linked to dysfunctions [45]. Indeed, the information processing system of writing is described as a capacity-limited system [46]: the more cognitive resources the individual allocates to transcription, the less he or she will have available for other required, high-level processes such as planning or texting [46,47].

Individuals must then develop their skills to become expert writers. During the early years of learning, the phoneme-to-grapheme conversion system and the graphomotor processes must be automated. These two processes are not automated in typically developing children until around 12 years of age [48]. From this period on, children begin to mobilize cognitive resources, not only for the management of the conversion system but also for high-level writing processes such as planning or revising the text [49]. Around 12–15 years of age, adolescents can more easily manage all of the processes involved in a writing task [49], but it is not until they are 16 years old that they can fully manage the planning processes [50]. In typical subjects, spelling processes become progressively more automatic with experience [48], which allows—within the framework of the theory of capacity for written production [46]—more resources to be allocated to higher-level processes.

This liberation of resources allows writers to resort to a knowledge transformation strategy (among others, [49,51,52]) or to knowledge crafting [53]. In order to respond to the constraint created by the multidimensional aspect of mental representations and the linear aspect of language, individuals resort to three planning strategies: knowledge telling, knowledge transforming, and knowledge crafting (among others, [49,51,52,53]). The knowledge-telling strategy corresponds to the step-by-step recovery of content or information, which is reformulated as it is recovered “without proceeding to an overall reorganization of the conceptual contents or the linguistic form of the text” ([54], p. 7). This type of planning is characterized by repetitive cycles of retrieval and formulation [46]. The knowledge transformation strategy calls for an effort to link two spaces: that of content (allowing the accessibility and organization of knowledge) and that of rhetoric (defining the constraints of goals given the situation) [49,51]. The organization of knowledge involves planning, which involves a great deal of domain, rhetorical, linguistic, and pragmatic knowledge about language. All these aspects are taken into consideration and constantly related to the linguistic task at hand. The speaker/writer must make the link between content knowledge, relating to domains and fields of study, and discourse knowledge. The latter includes syntactic aspects (e.g., word order in the sentence), text structures, and pragmatic knowledge (such as rules of language use, like punctuation). A third strategy is suggested, the knowledge-crafting strategy [53], which incorporates consideration of the interlocutor’s or reader’s mental representations. This type of planning is at play when the individual is able to use both the knowledge-telling strategy and the knowledge transformation strategy by taking into account the mental representations of the recipient of the message. Depending on the production context, the experience, and the age of the individuals, one strategy (telling, transforming, or crafting) may be more appropriate than another. Proceeding in the construction of a text step by step (knowledge-telling strategy) allows novice writers, especially children, to produce a text despite the fact that most cognitive resources are devoted to multiple demands of the writing activity (grapho-motor work). The knowledge transformation strategy can be mobilized when there has been automation of these low-level processes, requiring fewer cognitive resources, which are then available for this type of strategy. The knowledge-crafting strategy is mobilized rather late [53], when individuals become capable of ensuring a certain coherence between their ideas and their wording while taking into consideration the fact that their own mental representations differ from those of the recipients of their messages. The development of these strategies is strongly linked to the automation of certain processes, which implies, among others, the grapho-motor gestures and the spelling conversion system.

### 2.2. Role of Punctuation in the Written Task

Punctuation is a system specific to the written world and includes, in French, about fifteen graphic elements without phonemic counterparts [29,55]. This system is defined as visual signs of organization and presentation essential to written text [56], named by linguists “punctems” [29]. These marks have the main objective of marking the relationships between statements [57,58] and obey a hierarchy according to the degree of rupture they induce in the text: indent > point > comma > Ø [29,55]. Punctuation, like its analogues, anaphora and connectors, seems to serve three main functions: the prosodic function (specific to punctuation); the syntactic function; and the semantic function [32,56,57]. The prosodic function is related to the primary function of punctuation at the beginning of its integration into the sign system of writing, namely the marking of oral prosody [57,59]. Indeed, the hierarchy of signs corresponds to the length of the pause to be made in speech. The syntactic and semantic functions of punctuation serve respectively to delimit propositional units and to mark the degree of connection or rupture between constituents [30,31,33]. (Fayol [32], p. 24) presents punctuation as marking “the degree of binding (or breaking)” between adjacent propositions, i.e., the strength of the inter-event relations established in the writer’s mental model of the situation described. The more strongly two propositions are linked, the less they are separated by a punctuation mark.

While the punctuation system has an essential role in written production and participates in its cohesion, it seems to be one of the least studied linguistic paradigms [31,34,55,60]. Indeed, punctuation marks, like anaphora and connectors, are categorized among the cohesive marks of written production, which have real functional qualities in structuring the text [29]. Their production is determined during “textual linearization, an intermediary process between conceptual organization of planning and translation” [29], according to the models and work of Hayes & Flower [41] and Berninger & Swanson [48]. Linearization then appears as a high-level process, appearing early in the design of the message. It refers to the fact that the pre-linguistic representation of the message is rarely linear (as in narrative texts). Thus, “to produce in language is to apply”—in the mathematical sense of the term—“an organization that is not necessarily linear to another that is strictly linear” ([32], p. 24). This phenomenon of linearization implies linguistic elements that can be juxtaposed while their referents do not maintain a relationship in the mental representation of the message, and it is in this case that punctuation intervenes [32]. Linearization is therefore closely linked to coherence (cognitive representations associated with the written text produced) and cohesion (grammatical manifestations of coherence) [29,61,62].

To ensure cohesion and thus the marking of the hierarchy of cohesion marks, such as punctuation, the writer must carry out a hierarchical planning of the text, master the functional characteristics of the punctuation system (and of other cohesion marks), and take into account the knowledge of the addressee. It appears that these skills are acquired slowly and late [29]. The work linking punctuation management and textual planning reveals that punctuation itself is determined by the planning mode [30], a mode that depends on the expertise of the writer [49]. Efficient management of the linearization of the written message, then, requires procedural mastery of the plurifunctionality of these marks and of the possibility of considering the text as a whole, which requires a knowledge transformation strategy, a type of planning that appears later in the developmental process of written production (versus knowledge telling) [49], and which can be exploited, according to the theory of capacity-limited systems [46], if there has been automation of certain processes, such as orthographic conversion.

### 2.3. Impact of Dyslexia on Punctuation

For individuals with dyslexia, the conversion system is not fully automated, even in adulthood, and thus significant difficulties persist in written production [9,12,13,14,15,17,19,20,63,64,65,66,67,68,69] and textual production is impacted [47]. Dyslexia results in problems with composition, organization, writing, punctuation, and editing [24]. For instance, studies focusing specifically on spelling show that dyslexic students are reported to make more lexical [8,15,70], syntactic [9,14,15,16,17,63,64,67] and morphosyntactic [8,70,71] errors than non-dyslexic students. Horowitz and Breznitz [19] suggest that individuals with dyslexia have a deficit in the error detection mechanism, so they cannot identify all spelling errors and correct them. Some studies confirm that dyslexic students perform less well and less efficiently than non-dyslexic students [9,12,16,20,21]. Concerning lexical choices, some studies mention that dyslexic students may use unexpected or inappropriate vocabulary [8,68,72], produce fewer polysyllabic words than non-dyslexic students [8,16,68], and avoid using words for which they do not know the spelling, implying a preference for simpler vocabulary in writing and for words that are also simpler in spelling [8,16]. However, Hatcher et al. [17] (in English) and Mazur-Palandre et al. [14] (in French) contest these findings by stating that there are no differences in vocabulary level between dyslexic and non-dyslexic students. If students with dyslexia feel orthographic insecurity [12,15,20], they do not censor themselves regarding their linguistic choice: whether words have weak or strong orthographic consistency, whether they are long or short, it does not matter: dyslexic students use them [14]. As said before, another reported difficulty is punctuation. Few studies have addressed this linguistic paradigm in typical scriptors [31,34,55,60] and even fewer in dyslexics. Some studies analyze the written performance of students with dyslexia by examining several written indicators. For instance, Farmer et al. [16], analyzing narrative tasks and comparing students with dyslexia and students without, observed multisyllabic words, the number of spelling, lexical, and grammatical errors, the number of text construction errors, and errors of punctuation. The authors do not conclude that students with dyslexia have problems with punctuation, as some other studies suggest [73]. Another example is Connelly et al. [22], who measured the writing performance of students with dyslexia (compared to students without dyslexia). The study includes measures of transcription skills, including spelling and handwriting fluency, textual length indicators, lexical diversity, and punctuation skills. Tops et al. [38] compare the written texts of 100 students with dyslexia and 100 control students and include punctuation and capitalization in their measures, among spelling and morphosyntactic errors, words, and syntactic structures used. These two studies mention that dyslexic students have difficulties managing punctuation and capitalization, which is in line with other studies [22,23,24]. The authors conclude that there is a significant difference in the processing of punctuation between dyslexic and control students, which highlights a lack of management of this system of signs and, in particular, the period (full stop) and the comma [22,23,24,25]. 

This present paper focuses entirely on the production and management of punctuation by students with dyslexia in spontaneously written texts by comparing their texts to those of control students matched for age, gender, and academic level. The aim of this paper is also, first, to see whether students with dyslexia use the same inventory of punctuation as control students—do they use the same marks in the same proportion? Secondly, we want to see whether students with dyslexia handle the punctuation system in the same way that control students do. Our results are discussed in relation to the previous analyses in light of theories of written production and the types of planning they call for.

## 3. Hypotheses

The national and international literature attests to the difficulties that dyslexia creates for individuals. It has been clearly stated previously that the daily lives of these individuals, despite an increasingly early diagnosis and treatment, are strongly impacted [69]. Among the difficulties mentioned by dyslexic adults, language production, and particularly written production, remains a major difficulty [12,13,20,65,66], whether in lexical or grammatical spelling, in organizing a plan and a text, in managing punctuation, or in correcting themselves. These feelings have been confirmed by objective experiments and the results of studies revealing that adults with dyslexia have difficulties in lexical [8,14,15,70,71] and grammatical spelling [9,14,15,16,17,63,64,67], in managing punctuation [22,23,24,25], and in revision [9,12,16,20]. If students with dyslexia use the same linguistic inventory—for instance, they use the same syntactic structures [22,67], make identical lexical choices [14,22], and write texts of similar length as the students control [16,22,63], it seems that their texts show many more mistakes.

Moreover, according to the capacity theory [74], as envisaged in the case of written text production [46], cognitive resources are limited and are distributed among the different processes involved in text production according to their degree of automation. A largely automated low-level process thus requires few cognitive resources, whereas a less automated process requires more. Thus, a low level of automation of the orthographic system requires an important mobilization of resources to the detriment of high-level processes [46]. Adults with dyslexia do not appear to have fully automated the orthographic conversion system [9,12,13,14,15,17,19,20,66,67,68]. Thus, the cognitive resources allocated to transcription remain important, and therefore fewer resources are available for the other processes required, such as planning, text editing, or revision [46,47], and the final quality of the textual production is strongly impacted. Furthermore, as mentioned earlier, punctuation contributes to the cohesion of the text and constitutes a step of planning involving high-level processes. 

We then hypothesize that dyslexic students will show a different use of punctuation than control students:

**H1.**  *The dyslexic students use the same punctuation marks as the control students;*

**H2.**  *The dyslexic students use fewer punctuation marks than control students;*

**H3.**  *The dyslexic students realize more punctuation errors than control students.*

## 4. Methods

### 4.1. Participants

Data collection was carried out in the framework of three projects concerning the difficulties and needs of French students with dyslexia in higher education (the ETUDYS, DYS’R’ABLE, and FLEXIDYS projects (projects co-founded by the PEPS CNRS program, the LabEx ASLAN, the Ecole Normale Supérieure de Lyon, and the CNRS Laboratory ICAR (CNRS, Université Lyon 2, and Ecole Normale Supérieure de Lyon)). There were several steps in these projects: (1) two online questionnaires concerning difficulties and needs of students with dyslexia at the university, completed by 1454 students for the first one (analyses ran on the responses of 97 students with dyslexia and 97 control students matched in gender, age, and level of study), and 1472 for the second (analyses ran on the responses of 83 students with dyslexia and 83 control students matched in gender, age, and level of study); (2) a speech, language, and neuropsychological assessment (N = 30 students with dyslexia and 30 control students) (written language processing: ECLA 16+ battery [75] and “Le vol du PC” [76]; decoding: reading of isolated words (regular, irregular and pseudo-words), and texts (“Le vol du PC” and “L’Alouette”); spelling: dictation of isolated words (regular, irregular and pseudo-words) and text (ECLA 16+); reading comprehension: “Le vol du PC” text subtests; meta-phonological skills; neuropsychological tests: TAP-M [77]; visuo-attentional skills: TAP- Report Global test [78,79] (see EVADYS [80]); SIGL test [81]; visual search test (n-cancellation test, ECLA 16+); visual and auditory orientation tests (Visioner and Audioner [82]); perceptual reasoning (matrixes); short-term memory; and auditory-verbal working memory (number memory): tests from the Wechsler scales assessed. Results from this part of the protocol are reported in previous articles [13,83]; and (3) psycholinguistic tasks consisting of producing four text types (spoken narrative, written narrative, spoken expository, and written expository). 

The present paper focuses on the written psycholinguistic data of 21 students with dyslexia and 22 control students (Table 1). 

Both groups, students with and without dyslexia, are matched for gende, age (Table 1), and grade (Table 2).

All students with dyslexia diagnosed in childhood had associated dysorthographia and received speech and language therapy during their childhood/adolescence. At the time of data collection, only two students with dyslexia out of the 21 participants said that they were registered with the handicap service of their institution and thus had additional time to complete exams (none used specific digital tools or were following a remediation program at the time of collection). The students were all monolingual native French speakers; they all have an education in France and are supposed to have received the same education concerning writing and, more specifically, punctuation. They gave written consent to participate in the assessment and the psycholinguistic task, for which they received financial compensation. The exclusion criteria, verified during personal interviews (at the time of the assessment), excluded individuals with hearing or visual deficits or other disorders. 

### 4.2. Psycholinguistic Task: Data Collection

#### 4.2.1. Protocol: Textual Elicitation

After having filled out the questionnaire and passed the speech and neuropsychological assessments, the students completed the psycholinguistic task. They were asked to produce four texts on the theme of conflict between people, which results in four experimental conditions: oral narrative, written narrative, oral expository, and written expository text production tasks, following Berman and Verhoeven [84]. For the expository text, students were asked to produce a text on problems between people, discussing the theme by presenting their ideas as if in a school presentation. For the narrative text, students were asked to tell a personal story about a conflict they might have experienced. We asked participants to write by hand with a pen and paper, as they usually do in university settings, with no specific instructions about spelling, proofreading, etc., so as to collect the most naturalistic data possible. Moreover, they do not have any time limit to produce their text.

Data collection was conducted during two sessions with a one-week-long interval between them in order to avoid the effects of fatigue. Each subject had two appointments. During the first week, the project was rapidly presented to the participants, who then watched a video. It was a three-minute video film without words, depicting a variety of short scenes of conflict between people in a school environment. This video was specially created for the Spencer Project (responsible: Ruth Berman). Next, they had to produce a narrative or an expository text in both written and spoken modalities. Between the production of the written and spoken texts and in order to avoid transfer (word by word from one text to another), participants were asked to answer a questionnaire about written and spoken language (reading habits, relationship to the written word, etc.), given orally by the experimenters. During the second week, participants had to produce, in both written and spoken modalities, either a narrative or an expository text. Between the two texts, they also had to answer another questionnaire about written and spoken language. Students were divided into two test groups: half produced a written text followed by an oral text, and the other half produced an oral text followed by a written text. The production order was counter-balanced for text type as well: half of the participants produced an expository text first and then a narrative text, and the other half did the opposite. Students had no time limit and were allowed to take all the time they needed to write their texts and proofread them if they wanted. This present study focuses on written texts. 

#### 4.2.2. Written Data Exploitation

Written data were collected using digitizing tablets via the Eye and Pen© software [85] and transcribed according to CHILDES (https://childes.talkbank.org/, 9 July 2021) conventions with a transcriber for oral data and in Eye and Pen© for handwritten data, then exported into the CLAN software. The productions were divided into clauses—the clause being defined as a unit of meaning composed of a finite or non-finite verb and arguments—and terminal units (TU)—a unit made up of a main clause and all its dependent clauses. These two units have been shown to be appropriate for the evaluation of syntactic development [86,87]. For this study, our written text corpus consists of 86 text productions (43 expository and 43 narrative), which represent 2328 clauses (expository: 1089; narratives: 1239) and 1126 TU (expository: 515; narratives: 611). Table 3 shows the length of the texts according to text type and group.

ANOVA analyses show that differences in length between students with dyslexia and control students in the number of words (F_(1.39)_ = 0.089, *p* = 0.767), clauses (F_(1.39)_ = 1.842, *p* = 0.183), T units (F_(1.39)_ = 2.501, *p* = 0.122), and clauses per T unit (F_(1.39)_ = 0.773, *p* = 0.385) were not significant. Moreover, analyses reveal that differences in time duration (duration of written production) between the two groups are not significant (F = 2.07; *p* = 0.164 > 0.1).

#### 4.2.3. Analysis Categories

We list below the different punctuation marks found in our texts and provide for each the main conditions under which they should be used in written French texts, according to the norms [88,89]. Those norms will guide us in deciding where punctuation signs are expected—punctuation sites—and which punctuation sign is expected at this very place. The list is ordered according to the frequency of each sign produced (and therefore found) in control students’ texts (see Section 5.4). The given examples are extracted from the data. 

Capital (CAP). In our coding system, each punctuation sign is given a three-letter code: CAP stands for “capital”, POI for “point”, etc. Although capitals are not genuinely punctuation symbols, they do play a part in the punctuation system. A capital is expected on every word at the beginning of the sentence and therefore after every closure symbol (period, question mark, exclamation mark). We consider it erroneous in every case where the first word of the sentence is not capitalized. We also consider as erroneous every capitalized word that is not at the initial place of the sentence (unless it is a proper noun).

(1) Example of the correct utilization of the capital (student with dyslexia, n°2)

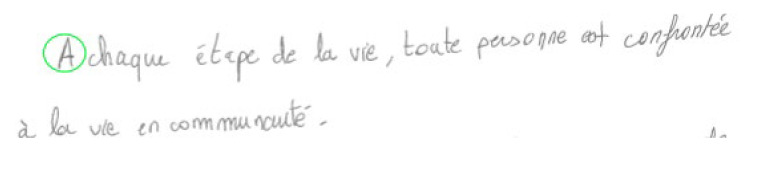

*“At every stage of life, every person is confronted with community life.”*

Period (PER). The period (full stop) is the main closure mark for sentences. It denotes the sentence is closed and its meaning is complete (2). As such, it differs from the colon, the semicolon, and the suspension points. The period is also a neutral mark with respect to the question mark and the exclamation mark. As the final mark of the sentence, it must be followed by a capitalization.

(2) Example of the correct utilization of the period (student without dyslexia, n°24)

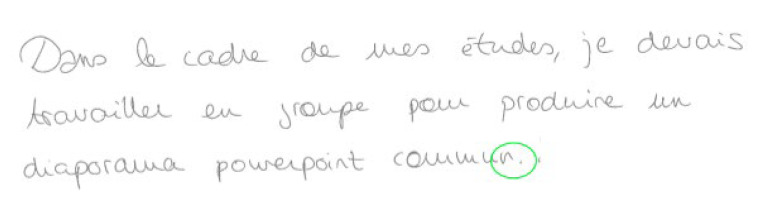

*“As part of my studies, I had to work in a group to produce a PowerPoint slideshow together.”*

Comma (COM). The comma is by far the most complex mark in the punctuation system. There are actually two commas. The first comma (comma plus, 88) works like coordinating conjunctions (like *and* or *or*) and helps to coordinate several elements of the same nature (verbs, adverbs, nouns, and adjectives) in an enumeration. The commas separate but connect items in a homogeneous set. The second comma functions only in pairs, just like dashes or parentheses, to separate a segment from the main syntactic organization of the sentence. It also marks any inversion in the canonical order of the sentence, e.g., when the sentence starts with an adverb instead of with the subject (3). In this usage, commas both deal with syntax and intonation.

(3) Example of the correct usage of the comma (student without dyslexia, n°36)

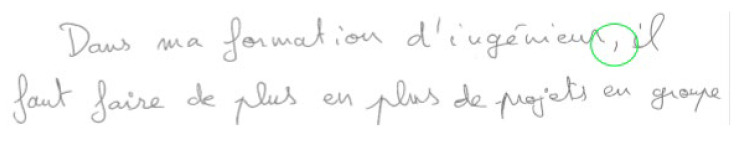

*“In my engineering training, I have to do more and more projects in groups”*

Parenthesis (PAR). Parentheses help to insert at any place in the sentence (one should avoid using them at the starting position) a verbal segment that should not be read in the current syntactic flow of the discourse and that is not mandatory for the global understanding. Each opening parenthesis must be followed by a closing one. Our coding system does not distinguish between the opening and closing symbols (4). We use a separate code for brackets (see below). We did not observe any use of braces.

(4) Example of the correct usage of parenthesis (student without dyslexia, n°51)

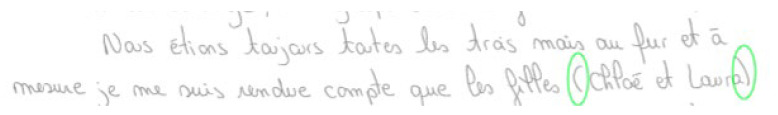

*“We were always the three of us but as time went by I realized that the girls (Chloe and Laura)…”*

Quote (QUO). The French punctuation system uses French quotes (« »). It uses English double quotes (“ “) when quoting has to be done inside French quotes. Quotes must be used for quotation (5), i.e., reporting into one’s discourse a segment from someone else. They are also a means to isolate or highlight a specific word or verbal segment from which the speaker wants to take a distance. We do not make any distinction between a French, English, single, or double quote, nor the opening and closing ones.

(5) Example of the correct usage of the quote (student without dyslexia, n°41)

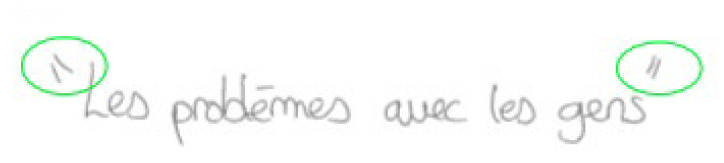

*“Problems between people…”*

Colon (COL). The colon can be used either (1) to introduce a conclusion, an explanation, etc. (2) an enumeration, (3) a quotation, an example, etc. It connects two (or more) segments in an asymmetric relationship: the following segments complete the preceding one. It is generally not followed by a capital. The capital is only expected when the following segment is a reported speech.

(6) Example of the correct usage of the colon (student with dyslexia, n°02)

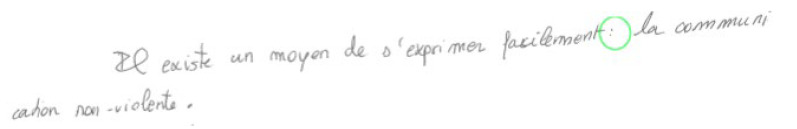

*“There is a way to express yourself easily: non-violent communication.”*

Exclamation mark (EXC). The exclamation mark denotes the end of the sentence together with the exclamative modality of the speaker (7). It manifests a personal, subjective reaction of the speaker in the enonciative situation. It may also be used inside the sentence, and in this very case, it should not be followed by a capital. Like the question mark, one may repeat the mark so as to enhance the affective dimension.

(7) Example of the correct usage of the exclamation point (student without dyslexia, n°51)

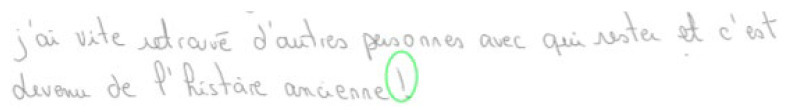

*“I quickly found other people to stay with and it became ancient history!”*

Suspension points (SUS). Suspension points (ellipsis) express incompleteness. They may denote a silence, an implicitation, or something that the speaker does not want to express (8). At the end of a sentence, suspension points are followed by a capital, but in other positions, it is not, since the following segment is the continuation of the preceding. Inside a sentence, it denotes a suspension of the discourse and should not be followed by a capital.

(8) Example of the correct usage of the suspension points (student with dyslexia, n°54)

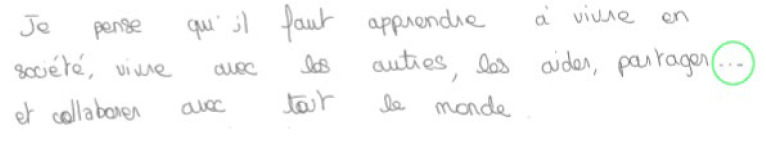

*“I think we have to learn to live in society, to live with others, to help them, to share... and to collaborate with everyone.”*

Dash (DAS). Dashes can be used alone or in a pair. The single dash is to be used in an enumeration sequence introduced by a colon and a line break. It then introduces each item of the sequence as a separate paragraph, closed with a semicolon. In pairwise usage, dashes are a stronger way to isolate a segment than parenthesis pairs or even commas. The dash should not be confused with the hyphen, which is not a punctuation mark. The hyphen belongs to the word itself and does not depend on the usage of this word in the sentence or the text.

(9) Example of the correct usage of the dash (student without dyslexia, n°24)

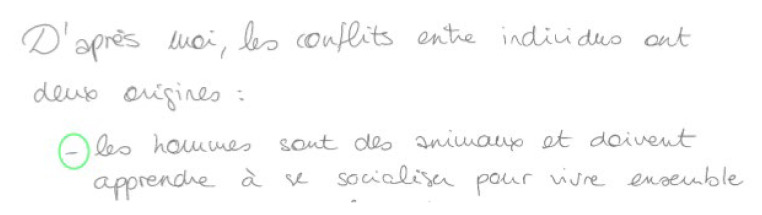

*“In my opinion, conflicts between individuals have two origins: - humans are animals and must learn to socialize to live together”*

Semicolon (SCO). Semicolons should be used to separate different long items in a sequence. It is also useful to maintain two autonomous but strongly linked propositions in loose or symmetric coordination (13). Contrary to the semicolon, there is no logical subordination of one element with respect to the other.

(10) Example of the correct usage of the semicolon (student without dyslexia, n°9)

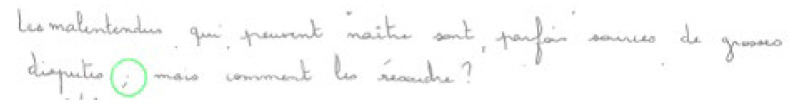

*“The misunderstandings which can arise are sometimes sources of big arguments; but how to solve them?”*

Question mark (INT, as an interrogation point). This symbol is the mark of interrogation and always denotes doubt, uncertainty, and puzzling (11). Like the exclamation mark, it is mostly a closure mark, followed by a capital. When used inside the sentence as a temporary question, it should not be followed by a capital.

(11) Example of the correct usage of the question mark (student with dyslexia, n°8)

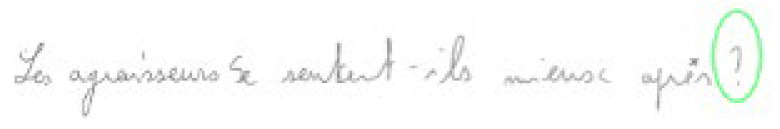

*“Do abusers feel better afterwards?”*

Slash (SLA). One may use the slash to mark alternatives between two terms (12).

(12) Example of the correct usage of the slash (student without dyslexia, n°51)

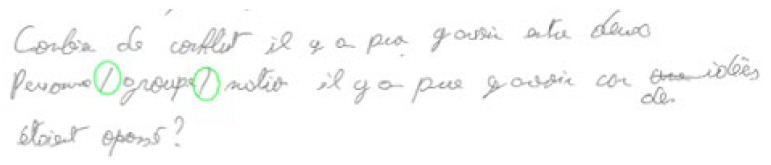

*“How many conflicts there may have been between two people/groups/nations there may have been because ideas were opposed”*

Bracket (BRA). Brackets ([]) may be used as parentheses inside a segment that is already between parentheses. The other usage is to mark the absence (ellipsis) or a modified portion of a quoted segment (13).

(13) Example of the correct usage of the bracket (student without dyslexia, n°9)

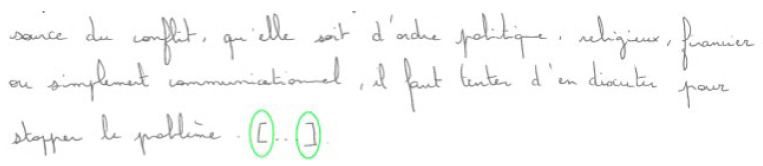

*“The source of the conflict, whether it is political, religious, financial or simply communicational, we must try to discuss it to stop the problem […]”*

The line break is also a punctuation mark, used to mark paragraphs, titles, and enumerations. In some contemporary usages, it may even replace the final point. Since we considered it more of a page formatting element than a punctuation one, we decided not to code this mark.

#### 4.2.4. Coding

Complying with the French punctuation norms and the methodological choices described above, we coded every punctuation mark (or absence of mark if the student forgot to add one) with a couple of codes (*x* and *y*), where *x* is the three-letter code of the mark produced by the student and *y* is the code corresponding to the mark expected at this place by the norms. For example, (COM; PER) encodes a place where the student wrote a comma (COM) but a period (PER) was expected. With the code Ø, we also note missing marks or add-ons (insertions). (Ø; COM) means that the student forgot to mark his/her text with a comma. Conversely, (COM; Ø) means that he or she wrote a comma at a place where the norms expected none.

This coding technique allows us to observe four different configurations:

- correct usage, when both codes *x* and *y* are identical;
- misses, when the first code is Ø;
- add-ons, when the second code is Ø;
- and confusion (or mismatch) when codes are different.

#### 4.2.5. Data Processing

In practice, coding was carried out in a visual manner by directly annotating the digitized PDF texts. Annotation consists of circling every punctuation site with a green circle when punctuation is correct, an orange circle when the produced mark is either unexpected (add-on) or incorrect (mismatch), and a red circle when a mark is missing. When a mark is missing or incorrect, one indicates inside the red or orange circle the code of the mark expected by the norms.

(14) Excerpt from a pre-coded dyslexic student’s text (n°01)

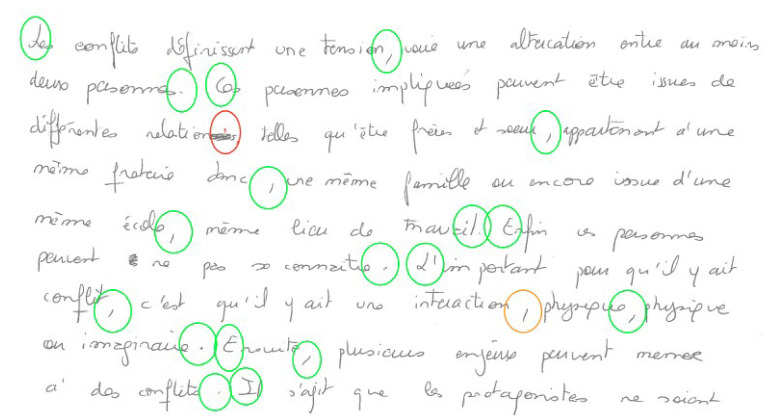


The final coding (with the couple (*x* and *y*)) is then reported at the right place in the CLAN transcript, allowing us to perform, in the near future, qualitative error analysis taking into account the context of production. For the current quantitative study, contextual information has not been exploited. We simply extracted from CLAN every coding couple and observed the distribution of punctuation marks and of the potential errors. 

Inter-judge method. The data were transcribed, pre-coded, and cut into clauses by one transcriber, and then proofread and corrected by a second transcriber. A third transcriber checked all clause and T-unit breakdowns. Problematic cases were discussed in a meeting, and a decision was made by the team. For the punctuation pre-coding, two coders pre-coded all the data (circle markers in the pdf). Problematic cases were discussed, and a decision was made. Then, a third coder coded all the data in the CLAN software with an automated code file, which limited errors. Finally, two other coders coded 10% of the data in order to test the reliability of the coding; the agreement between the coders was 94.92%.

## 5. Results

### 5.1. Introduction to Results

Four analyses were performed. We first wanted to get an overview of the usage of punctuation marks in both groups (students with dyslexia and control students). The objective was to observe the usage of each punctuation mark, sketch where the main differences could be, and perform the right statistical tests. Confusion matrices are the tool chosen for this task. This task is purely qualitative and cannot estimate whether any difference is significant or not.

Then we tested whether students with dyslexia use fewer punctuation marks in their texts (Hypothesis H2). The amount of punctuation is evaluated with respect to the text length (number of words).

The third step is the comparison between inventories (Hypothesis H1). We test whether students use the same set of marks as control students, in the same proportions.

Finally, we perform an exhaustive and systematic comparison of every punctuation mark in order to test whether some punctuation signs are harder to handle than others (Hypothesis H3). 

### 5.2. Confusion Matrix

#### 5.2.1. Presentation

Confusion matrices are square arrays counting in each cell (row *x*, column *y*) the number of couples (*x* and *y*) observed in the data, i.e., the number of times a participant used the punctuation mark *x* instead of the mark *y*. When no sign was expected, we counted it in the first column (named ADD, line *x*). When a participant failed to insert the mark *y*, we counted it in the first line (named MISS, column y). 

Confusion matrices help to count and observe the distance between usage (produced punctuation) and norms (expected punctuation). The higher the numbers we have in the diagonal, the more punctuation usage complies with the norms. Inversely, large numbers outside the diagonal (and in the first row and column) indicate where the main confusions are and which categories (marks, rules) are the most difficult to handle.

We gathered all couples from 44 texts from the control group and 42 texts from students with dyslexia. To observe the main trends, we focus on numbers greater than 10.

#### 5.2.2. Control Group

**Figure 1:**
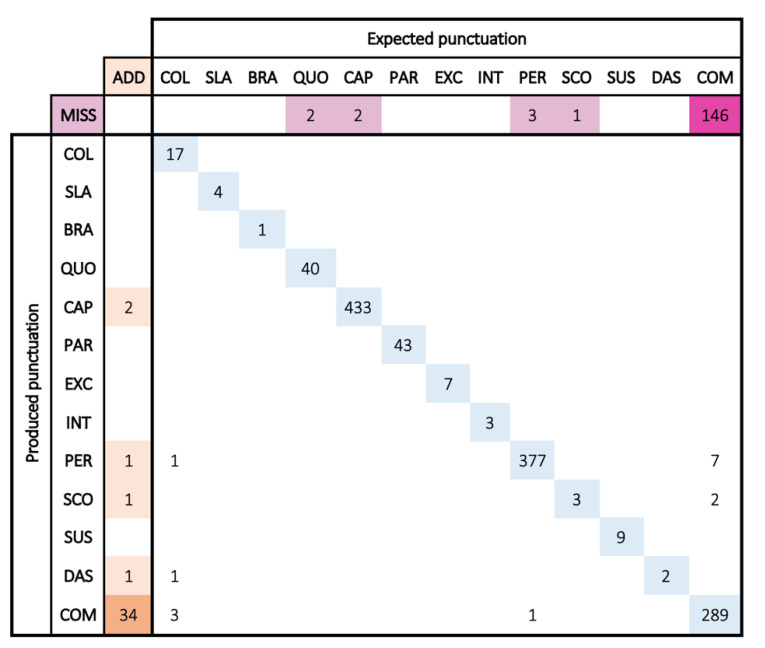
Control group confusion matrix: in the rows, how much each symbol was produced (or missed); in the columns, how many times it was expected (or added) (see Section 5.2.1 for interpretation guidelines).

Inspecting the control group confusion matrix (Figure 1) is a good way to obtain an overview of the main difficulties of the punctuation system. The diagonal numbers are quite high, and the array is very sparse, showing that the rate of correct punctuation should be very high. The punctuation system is well mastered. 

Regarding the missing marks (row MISS), they concern only one category: commas (146 misses). Control students mainly miss commas, but in a very large quantity. Regarding added marks (column ADD), there are fewer errors than misses, but also commas (36). Confusion errors (mismatches) are located in the central white zone, outside the diagonal. The numbers are very low.

The punctuation system is very well mastered, except for the use of the comma.

#### 5.2.3. Students with Dyslexia

**Figure 2:**
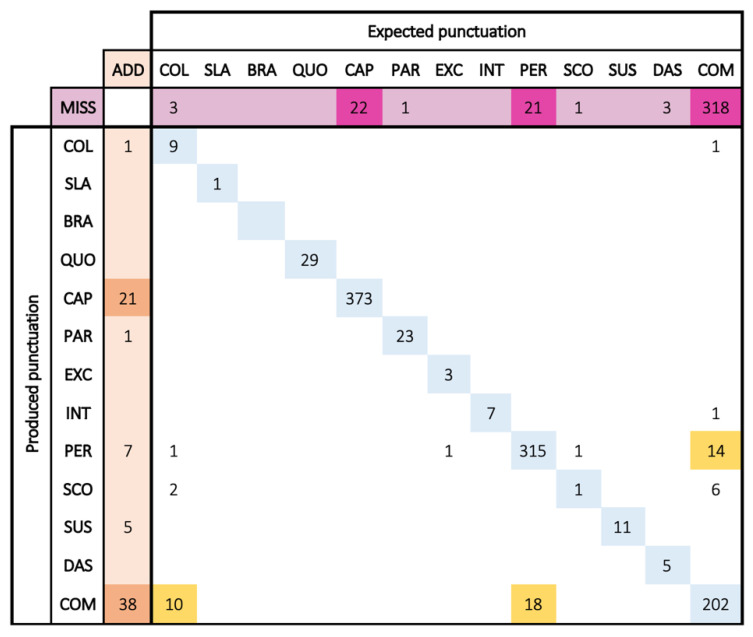
Dyslexic group confusion matrix: in rows, how much each symbol was produced (or missed); in columns, how many times it was expected (or added) (see Section 5.2.1 for interpretation guidelines).

This confusion matrix (Figure 2) is very similar to that of the control group. The diagonal numbers are still high, but a little less so.

On the first row (MISS), there are more missing marks, especially with commas (like the control group) but also with capitals (CAP) and periods (PER). In the first column (ADD), we observe that dyslexic students insert unexpected commas (like control students) but also unexpected capitals.

Dyslexic students also make more mismatches, which are highlighted with the yellow color. This confusion lies between commas and periods (18 and 14 errors), which may mean they have difficulties handling sentence closures. There is as well a confusion between colon and comma (10 errors). Confusions concern the same marks as in the control group but to a greater extent. 

#### 5.2.4. Conclusions

If we focus on the most frequent errors (more than 10 all over the group), there are only 2 sources of errors in the control group (missed and added commas), compared to 8 sources for students with dyslexia: missing commas, periods, and capitals; added commas and capitals; and mismatches on commas, periods, and colons. 

We need to focus our tests on those specific points to check whether those differences are significant or not.

### 5.3. General Proportion of Punctuation in Texts

The punctuation rate (PR) is calculated on each text, giving the average number of punctuation marks (correct or not) every 100 words. With a Welch test (a generalization of the T-test for groups of different sizes), we test whether the punctuation rate significantly differs between the group of students with dyslexia and the control students.

As shown in Table 4 below, the punctuation rate for students with dyslexia is two points less than for control students (13.7 against 15.7), and this difference is significant (T = 2.58; Df: 37.5; *p* = 0.014 < 0.05). We reject the null hypothesis and may claim that students with dyslexia produce significantly *less* punctuation than control students (the H2 hypothesis is confirmed).

### 5.4. Inventory

For each student, we evaluate the proportion of each punctuation mark (capital, point, etc.) with respect to the total of punctuation produced in the text. We can then observe whether a given punctuation mark is less used in a given group. A bilateral Welch test is applied for every punctuation mark.

As shown in Table 5 below, the most frequent punctuation marks are capitals (around 35%), periods (around 30%), and commas (around 24%). Those three categories cover more than 90% of punctuation production.

Welch tests conclude that there is no significant difference between the two groups for any punctuation mark. We maintain the null hypothesis: students with dyslexia use the same inventory of punctuation marks as control students (H1 is confirmed) and in comparable proportions.

### 5.5. Punctuation Errors 

#### 5.5.1. Global Error Rates

For each student text, we count the number of erroneous punctuation marks (missing, inserted, or confused). Four error rates are then calculated, corresponding to those amounts per 100 words of text. We compare each error rate between both groups with a bilateral Welch test.

As shown in Table 6 below, students with dyslexia make significantly more errors than control students (5.7 vs. 2.4 errors per 100 words). This difference is observed for any type of error: missing marks (4.1 vs. 1.9), inserted marks (0.9 vs. 0.4), and confusions (0.7 vs. 0.1). This confirms our third and last hypothesis (H3).

The following sections will develop those comparisons, taking a close look at each punctuation mark, in order to check whether some specific marks may be identified as potential causes of difficulties. This would also help to confirm (or disqualify) the observations made on confusion matrices (Section 5.2.4).

#### 5.5.2. Missing Punctuation Marks

For each punctuation category (e.g., comma), we count every time such a punctuation mark (comma) was missing, i.e., it was expected at a place where no punctuation mark was present. The missing rate corresponds to the ratio of this number divided by the amount of the same punctuation mark (comma) as expected by the norm in this text (including confusions and correct uses). For some categories, marks are quite rare and may even be absent in many texts. Such rates may therefore not be calculated for every text. We summarize in Table 7 the missing rates and the number of texts concerned by this measure.

When we analyzed confusion matrices (Section 5.2.4), we mentioned three potential differences concerning missing punctuation marks: commas, capitals, and periods. With our statistical tests reported in Table 7 above, two differences are confirmed: commas and periods. Concerning capitals, the difference is too weak (*p* = 0.0572).

#### 5.5.3. Mismatches between Punctuation Marks

For each punctuation category (e.g., comma), we count every time such a punctuation mark (comma) has been confused, i.e., it was expected at a place where another punctuation mark has been written (e.g., a point). The confusion rate corresponds to the ratio of this number divided by the amount of the same punctuation mark (a comma) as expected by the norm in this text (including missing and correct marks). For the same reasons as above, such rates may not be calculated on every text. We summarize in the following Table 8 the confusion rates and the number of texts concerned by this measure.

When we analyzed confusion matrices (Section 5.2.4), we mentioned three potential differences concerning confusions: commas, periods, and colons. With our statistical tests reported in Table 8 above, only one difference is confirmed: periods. Concerning the colons, despite the high level of errors (55% for students with dyslexia), the difference is not significant (*p* > 0.1). This type of punctuation is difficult to handle for students of both groups.

#### 5.5.4. Added Punctuation Marks

Finally, for each punctuation category (e.g., comma), we count every time such a punctuation mark (comma) has been added or inserted, i.e., at a place where no punctuation mark was expected by the norms. The insertion rate corresponds to the ratio of this number divided by the amount of the same punctuation mark (a comma) produced throughout this text (including confused and correct marks). For the same reasons as above, such rates may not be calculated on every text. We summarize in the following Table 9 the insertion rates and the number of texts concerned by this measure.

When we analyzed confusion matrices (Section 5.2.4), we mentioned two potential differences concerning confusions: commas and capitals. With our statistical tests reported in Table 9 above, only one difference is confirmed: capitalization. Concerning commas, despite the high level of errors (14.8% for students with dyslexia), the difference is not significant (*p* > 0.1). This type of punctuation is difficult to handle for students of both groups.

#### 5.5.5. Summary of Results Concerning Punctuation Errors 

As we said in Section 5.5.1, our hypothesis H3 has been confirmed: students with dyslexia commit significantly more errors than control students, and this difference is also confirmed regarding missing punctuation, inserted punctuation, and mismatch errors.

We also confirmed half of the observations made on the confusion matrix regarding specific punctuation signs. Students with dyslexia do show specific or enhanced difficulties when handling periods, commas, and capitals (which are also the most frequent punctuation marks). More precisely, their main weaknesses involve the following four errors: missing periods, missing commas, inserted capitals, and confusion regarding periods (they use too many commas instead of periods). Regarding other error configurations, no significant difference was observed.

## 6. Discussion 

The main goal of this paper was to deepen knowledge of the use and management of punctuation by students with dyslexia. As mentioned before, some international studies conclude that they can experience difficulties with this linguistic paradigm by making many errors [22,23,24,38]. In this context and given the lack of studies on the management of punctuation in adults with dyslexia, it seemed important to fill this gap in order to help them better understand their differences in learning and manage their difficulty in writing. We also proposed three general hypotheses: H1. The dyslexic students use the same punctuation marks as control students; H2. The dyslexic students use fewer punctuation marks than control students; and H3. The dyslexic students realize more punctuation errors than control students.

The analyses reveal that dyslexic students possess the same inventory of punctuation marks as control students (H1: confirmed). For both populations, the use of punctuation is mainly focused on the capital letter (which, in our data, is treated as a punctuation mark), the period, and the comma, confirming previous works for French scriptors [31,90,91]. The use of other punctuation marks such as parentheses, colons, suspension points, or the exclamation mark remains very marginal. That being said, if dyslexic students use the same inventory of punctuation marks as control students, the analyses reveal that they use, however, less of them (H2: confirmed). Finally, dyslexic students make more punctuation errors than controls (H3: confirmed): they make more confusions of signs (especially between the period and the comma), more omissions of commas and periods, and finally more additions of capital letters. We have to take into consideration that these marks (capital, comma, and period) are the most frequent in the written text of our population (Table 4). Moreover, these punctuation errors persist despite proofreading. Indeed, two previous studies on the same dataset revealed that both groups do revise their texts. There is not a significant difference concerning the proportion of revision in the text between students with dyslexia and control students. Students with dyslexia realize almost as many revisions as control students, but their revisions mostly concern lexical and grammatical spelling and marginally punctuation [12,20]. Note that the difference between the number of words per text for students with dyslexia and control students is not significant. Additional analyses of chronometric measures (production and pause durations, flow, speed, etc.) are underway and may complement these results.

The following discussion addresses specifically those punctuation marks. These results may reveal a limitation in the use and management of punctuation, which may be an indicator of a deeper problem linked to higher-level processes of written production.

Indeed, the use of the period and comma plays a role in the cohesion of a text and comes into play as soon as the text is linearized, which requires procedural learning of the multifunctionality of these marks, and more specifically the comma, and the possibility of considering the text as a whole [30]. According to the model of Hayes and Flower [41], punctuation intervenes during textual linearization [29], an early step in the written task. Management of punctuation is a late acquisition [29] and calls for a high-level planning strategy: knowledge transformation (versus knowledge telling) [49]. Students with dyslexia continue to allocate cognitive resources to low-level processes (such as transcription and, more precisely, orthographic conversion); therefore, they do not have sufficient available cognitive resources for high-level processes such as planning or linearization, implying punctuation [46,47]. This has a direct consequence on the use of the period and comma in their written texts, with an impact on every sentence of the text.

Its mismanagement could also reveal problems of macro-structural and enunciative semantics, since point and comma mark the degree of connection or rupture between constituents [30,31,32,33,57,58,91] and thus allow the hierarchization of ideas and therefore of sentences. The period is usually used to separate less linked constituents, whereas the comma is used for more linked constituents. The errors in the use of the period and comma of dyslexic students, and specifically the confusion between period and comma, can reveal a problem of hierarchization of the constituent. All the more so, since the degree of link between constituents is obviously established in the mental model, an early stage of textual production. 

## 7. Conclusions 

To conclude, we can say that our results suggest that having faulty usage of periods and commas is indicative of deeper difficulties, such as the linearization of the message, the mobilization of high-level planning strategies (such as knowledge transformation strategies or knowledge crafting strategies), and the hierarchization of information. Nonetheless, control students also show a restricted use of punctuation, reduced to the period and the comma, and also make many errors on commas (but still fewer than students with dyslexia). We can associate these limits of their expertise with the teaching of punctuation in France. The teaching they receive often boils down to a syntactic approach (“the sentence begins with a capital letter and ends with a period”) that is unable to account for usage [91].

To finish, it seems necessary to point out the limitations of this current study. The major limitation of this study is that we use the category of dyslexia without being able to distinguish the underlying deficits. There are relatively few studies that have characterized developmental dyslexia in adults, and it seems very difficult to identify precisely the type of deficits involved in adults [13]. Habib [4] describes three profiles of dyslexia for children, linked to several types of disorders: linguistic, attentional, or motor, which may suggest potentially different mechanisms of origin and specific clinical impacts. Moreover, given the lack of studies in the field of dyslexia in adulthood, we are forced to build upon the research on dyslexia conducted during childhood. Another limit is the fact that, even if all the participants in this present study were educated in France and thus received the same instruction concerning punctuation, they may have a variable level of knowledge about this paradigm. Thus, we could have considered a questionnaire or a pre-test before the task to get information about their knowledge of punctuation. It is not always planned in semi-experimental psycholinguistic studies on spontaneous written data to include such pre-tests.

## 8. Perspectives

In this paper, we study punctuation, one of the linguistic tools to ensure textual cohesion, and we conclude that the difficulties of students with dyslexia in managing punctuation can reveal deeper difficulties in linking with the linearization of the message and hierarchization of information. But additional studies could provide more precise answers to many questions. First, in order to consolidate our conclusion concerning the linearization and hierarchy of constituents, it seems necessary to collect a much larger set of data. This will allow us to obtain more occurrences of all the punctuation marks, including the less frequent ones (such as the semicolon, parenthesis, and colon). Second, we plan to complete the study with one covering two other tools ensuring textual cohesion: anaphora and connectors. Third, we can analyze punctuation from an online perspective, taking into account online indicators such as pauses and writing speed. Previous studies show that pauses are of different lengths depending on the punctuation mark realized [60,92]. Foulin et al. [92], for instance, found that pauses associated with paragraphs are longer than those corresponding to the period and capital, which are longer than those associated with the comma. Moreover, the authors also show that it is necessary to distinguish pauses that precede or follow the punctuation marks. Finally, the authors show that the need to develop a mental model and to linearize the message mobilizes cognitive resources, which manifest themselves, among other things, in variations of certain temporal parameters of production management: in particular, the duration of pauses.

## Figures and Tables

**Table 1:** Description of participants in the psycholinguistic task.

	Students with Dyslexia	Control Students
Mean age	21.7	21.8
Range	18.1–28.5	18.1–28.9
Age standard deviation	2.8	3
Total number of subjects	21	22
According to gender	9 women/12 men	10 women/12 men

**Table 2:** Description of participants in the psycholinguistic task.

	Students with Dyslexia	Control Students
Licence	15	15
Master	4	5
PhD	1	1
Other	1	1

**Table 3:** Length indicators for written texts according to text type and group.

	Expository Texts		Narrative Texts	
	Students with Dyslexia	Control Students	Students with Dyslexia	Control Students
The mean number of words per text	198.2 (101)	181.1 (139)	207 (131)	181 (112)
The mean number of clauses per text	25.8 (12.7)	25 (17)	30.7 (20)	27 (16)
The mean number of TU per text	12 (5)	12 (9.4)	14.7 (9.9)	13.8 (8.2)
The mean number of clauses per TU	2.1 (0.5)	2.2 (0.4)	2.1 (0.3)	2.04 (0.5)
The mean duration of production (in minutes)	13.85 (8.60)	11.02 (8.16)	11.77 (7.60)	9.01 (4.94)

**Table 4:** Punctuation rate in both groups (Welch test).

	Students with Dyslexia	Control Students
Punctuation rate	13.7 (2.9)	15.7 (2.2)

T: 2.58; Df: 37.5; *p* = 0.0139; S.

**Table 5:** Proportion of marks in a given punctuation category (mean, SD, and Welch *p*-value: NS = non significant/S = significant).

	Students with Dyslexia	Control Students	*p*-Value
Capital	36.8 (6)	34.9 (4.4)	0.2388 (NS)
Period	30.9 (7.3)	30.5 (5.5)	0.8373 (NS)
Comma	23.7 (9.5)	24.8 (7.3)	0.6585 (NS)
Parenthesis	2.1 (3.9)	3.1 (3.4)	0.4147 (NS)
Quote	2.2 (3.3)	2.7 (3.4)	0.6091 (NS)
Colon	1 (1.7)	1.2 (1.8)	0.743 (NS)
Exclamation mark	0.1 (0.6)	0.9 (2.3)	0.1773 (NS)
Suspension points	1 (2.1)	0.8 (1.5)	0.7007 (NS)
Dash	0.4 (1.1)	0.4 (1.5)	0.9867 (NS)
Semicolon	0.8 (2.1)	0.4 (0.9)	0.3563 (NS)
Question mark	0.8 (1.7)	0.2 (0.4)	0.1032 (NS)
Slash	0.1 (0.5)	0.2 (1)	0.6899 (NS)
Bracket	0 (0)	0.1 (0.4)	0.3287 (NS)

**Table 6:** Average error rates concerning punctuation in both groups (mean, SD, and Welch *p*-value: NS = non significant/S = significant).

	Students with Dyslexia		Control Students		*t*-Test
	N	Mean (S.D)	N	Mean (S.D)	*p*-Value
Missing punct.	21	4.1 (1.8)	22	1.9 (0.9)	<0.001 (S)
Inserted punct.	21	0.9 (0.5)	22	0.4 (0.4)	<0.001 (S)
Confusions	21	0.7 (0.5)	22	0.1 (0.3)	<0.001 (S)
Total	21	5.7 (1.7)	22	2.4 (0.9)	<0.001 (S)

**Table 7:** Missing rates for each category of punctuation (mean, SD, and Welch *p*-value: NS = non significant/S = significant).

	Students with Dyslexia		Control Students		*t*-Test
	N	Mean (S.D)	N	Mean (S.D)	*p*-Value
Capital	21	3.8 (8.0)	22	0.3 (1.0)	0.0572 (NS)
Period	21	5.6 (7.8)	22	1.0 (2.9)	0.0172 (S)
Comma	21	56.3 (22.2)	22	32.1 (14.4)	0.0002 (S)
Parenthesis	8	1.8 (5.1)	12	0.0 (0.0)	0.3506 (NS)
Quote	9	0.0 (0.0)	10	4.2 (9.0)	0.1773 (NS)
Colon	13	9.2 (18.9)	10	0.0 (0.0)	0.1039 (NS)
Excl. mark	2	0.0 (0.0)	4	0.0 (0.0)	no error (NS)
Susp. points	6	0.0 (0.0)	6	0.0 (0.0)	no error (NS)
Semicolon	2	50 (70.7)	4	25 (50)	0.7098 (NS)
Quest. mark	5	0.0 (0.0)	3	0.0 (0.0)	no error (NS)

Bracket, slash, and dash are too rare to be reported in this table.

**Table 8:** Confusion rates for each punctuation category (mean, SD, and Welch *p*-value: NS = non significant/S = significant).

	Students with Dyslexia		Control Students		*t*-Test
	N	Mean (S.D)	N	Mean (S.D)	*p*-Value
Capital	21	0.8 (2.7)	22	0.0 (0.0)	0.1861(NS)
Period	21	4.4 (4.9)	22	0.1 (0.5)	0.0007 (S)
Comma	21	4.1 (7.8)	22	2.0 (5.5)	0.3216 (NS)
Parenthesis	8	0.0 (0.0)	12	0.0 (0.0)	no error (NS)
Quote	9	0.0 (0.0)	10	0.0 (0.0)	no error (NS)
Colon	13	55.0 (46.0)	10	26.7 (37.0)	0.1167 (NS)
Excl. mark	2	50.0 (70.7)	4	0.0 (0.0)	0.5000 (NS)
Susp. points	6	0.0 (0.0)	6	0.0 (0.0)	no error (NS)
Semicolon	2	25.0 (35.4)	4	0.0 (0.0)	0.5000 (NS)
Quest. mark	5	0.0 (0.0)	3	0.0 (0.0)	no error (NS)

Bracket, slash, and dash are too rare to be reported in this table.

**Table 9:** Insertion rates for each category of punctuation (mean, SD, and Welch *p*-value: NS = non significant/S = significant).

	Students with Dyslexia		Control Students		*t*-Test
	N	Mean (S.D)	N	Mean (S.D)	*p*-Value
Capital	21	5.5 (6.0)	22	0.2 (0.8)	0.0007(S)
Period	21	1.5 (3.5)	22	0.1 (0.5)	0.0872 (NS)
Comma	21	14.8 (17.3)	22	7.8 (8.4)	0.1016 (NS)
Parenthesis	8	1.8 (5.1)	12	0.0 (0.0)	0.3506 (NS)
Quote	9	0.0 (0.0)	10	0.0 (0.0)	no error (NS)
Colon	7	14.3 (37.8)	9	0.0 (0.0)	0.3559 (NS)
Susp. points	6	26.7 (29.4)	6	0.0 (0.0)	0.0772 (NS)
Dash	4	0.0 (0.0)	3	33.3 (57.7)	0.4226 (NS)
Semicolon	4	0.0 (0.0)	4	8.3 (16.7)	0.3910 (NS)
Quest. mark	5	0.0 (0.0)	3	0.0 (0.0)	no error (NS)

Bracket, slash, and exclamation mark are too rare to be reported in this table.

## Data Availability

The data are not publicly available.
